# Predictive value of immune inflammation index and systemic inflammation response index for contrast-induced acute kidney injury in endovascular treatment for acute ischemic stroke

**DOI:** 10.1097/MD.0000000000043642

**Published:** 2025-08-01

**Authors:** Derya Ozdogru, Abdullah Yildirim, Gulcin Yenice, Onur Kadir Uysal, Ilker Ozturk, Onur Kaypakli, Durmus Yildiray Sahin, Zulfikar Arlier

**Affiliations:** a Department of Neurology, Adana City Training and Research Hospital, University of Health Sciences, Adana, Turkey; b Department of Cardiology, Adana City Training and Research Hospital, University of Health Sciences, Adana, Turkey; c Department of Cardiology, Hatay Mustafa Kemal University, Hatay, Turkey

**Keywords:** contrast-induced acute kidney injury, endovascular treatment, systemic immune inflammation index, systemic inflammation response index

## Abstract

Endovascular treatment (EVT) is the treatment method for acute ischemic stroke (AIS) caused by large vessel occlusion. Contrast-induced acute kidney injury (CI-AKI) is a relatively common complication. In this study, we aimed to investigate the incidence and possible risk factors of CI-AKI including systemic inflammation response index (SIRI) and systemic immune inflammation index (SII) in patients undergoing mechanical thrombectomy. We retrospectively analyzed patients who underwent EVT for acute cerebral large vessel occlusion between January 1, 2021, and March 31, 2024. Before the intervention, blood was drawn from the antecubital vein and collected in the appropriate tubes. Serum levels of neutrophils, lymphocytes, platelets, hemoglobin, CRP, and albumin were measured, alongside lipid profiles and liver and kidney function parameters. An increase in serum creatinine of 0.5 mg/dL or more than 25% in the measurements 48 to 72 hours after EVT compared to before was defined as CI-AKI. SII was calculated with the formula of: peripheral (platelet count × neutrophil count)/ lymphocyte count; SIRI was calculated with the formula of: (neutrophil count × monocyte count)/ lymphocyte count. Their predictive value for CI-AKI occurrence was compared. CI-AKI was detected in 54 (19%) of the 281 patients included in the study. In terms of clinical parameters, baseline NIHSS, postthrombectomy, cerebral edema, hemorrhagic transformation, recanalization time, discharge mRS and contrast amount were significantly higher in patients with CI-AKI. SII exhibited the highest discriminative performance in predicting CI-AKI in patients undergoing mechanical thrombectomy, with a threshold of > 1784 (AUC = 0.710, 95% CI: 0.631–0.788, *P* < .001, sensitivity 42.6%, specificity 89.0%) followed by SIRI (AUC = 0.652, 95% CI: 0.576–0.728, *P* < .001, sensitivity 88.9%, specificity 37.9%). SII and SIRI were found to be significantly higher in patients with CI-AKI. These scores may be new promising low-grade inflammatory indicators for predicting CI-AKI development.

## 1. Introduction

Endovascular treatment (EVT) is the frontline treatment method for acute ischemic stroke (AIS) due to large vessel occlusion (LVO).^[1]^ Although it is very beneficial, EVT is associated with kidney dysfunction due to contrast agents. Contrast-induced acute kidney injury (CI-AKI) is a relatively common complication.^[2]^ While usually transient and reversible, it sometimes is severe enough to require dialysis. Risk factors for CI-AKI include advanced age, diabetes, previous renal dysfunction, decreased renal perfusion due to hypovolemia or heart failure, and the use of high amounts of contrast agent or ionic hyperosmolar contrast medium.^[2]^

CI-AKI was observed in approximately 7% of cases in a meta-analysis of patients with AIS treated with EVT.^[3]^ Subjects with end-stage renal failure are excluded from this endovascular treatment.^[3]^ The incidence and etiology of CI-AKI after EVT have not been adequately evaluated, but there are some studies demonstrating the association of CI-AKI occurrence with poor prognosis.^[4,5]^ Therefore, determining the risk factors underlying the development of CI-AKI after EVT is critically important.

Inflammation is a key determinant in the pathophysiology and outcome of AIS. Inflammatory biomarkers may be helpful in terms of predicting mortality and functional outcomes. The neutrophil-to-lymphocyte ratio (NLR) and platelet-to-lymphocyte ratio (PLR) reflect oxidative stress-related inflammation that have a key role for the occurrence and progression of CI-AKI. In particular, NLR predicts a high risk of CI-AKI development after cardiovascular interventions and surgery.^[6]^ Both thrombocytosis and lymphocytopenia are associated with the degree of systemic inflammation, and PLR is a marker that includes both hematological indices.

High PLR value have shown to be associated with CI-AKI in several studies.^[7,8]^ The systemic inflammation response index (SIRI) is based on monocyte, neutrophil, and lymphocyte cell counts, and the systemic immune inflammation index (SII) is based on neutrophil, platelet and lymphocyte counts.^[9,10]^ SIRI and SII are used to determine prognosis, especially in cancer patients, but only a few studies have investigated their role in AIS. To the best of our knowledge, this is the first study to investigate the relationship between SII and SIRI and the occurrence of CI-AKI. The aim of our study was to investigate the incidence and possible predictors of CI-AKI in patients undergoing EVT. Additionally, we planned to reveal how the prognosis is affected in patients with CI-AKI, as well as the rates at which complications such as cerebral edema and hemorrhage develop.

## 2. Methods

### 2.1. Study participants and endovascular procedure

We retrospectively analyzed patients who underwent mechanical thrombectomy (MT) for acute cerebral LVO between January 1, 2021, and March 31, 2024. The study has been approved by the Health Sciences University, Adana City Education and Research Hospital Clinical Research Ethics Committee (date: December 10, 2024, nu: 170). Individual consent was not obtained in this single-center cross-sectional study because of retrospective analysis of the data. All consecutive patients with AIS who undergo MT were included in the study. The decision to perform MT in the treatment of these patients was made according to the American Heart Association/American Stroke Association 2019.^[11]^ Accordingly, patients had Alberta stroke program early computed tomography scores (ASPECTS) ≥ 6, National Institute of Health Stroke Scale (NIHSS) ≥ 6, and large or medium vessel occlusion. There are 2 main methods in EVT. These are thrombectomy with retractable stents or thrombectomy based on thrombo-aspiration with large catheters (ADAPT). The second approach uses combined methods. The method to be applied was chosen here during the procedure based on the patient’s details. Magnetic resonance imaging (MRI) and computed tomography (CT) were used for the diagnosis of AIS. Contrast-enhanced computed tomography angiography was used when MRI could not be performed or the diagnosis of LVO was unclear. MT was performed with or without the combination of intravenous recombinant tissue plasminogen activator (IV rt-PA). Iohexol (Omnipol® 350; Polifarma, Istanbul) was used as a nonionic low osmolar contrast agent during MT. The amount of contrast medium was estimated according to the angiography image count.

We excluded patients with acute renal failure or end-stage renal failure at presentation, active infection, severe liver disease, known malignant diseases, chronic steroid treatment due to autoimmune diseases and contrast agent exposure in the last 2 weeks (except for the need for contrast-enhanced CT for the diagnosis of LVO).

The outcome measures included the time to recanalization and the presence of cerebral edema and hemorrhage on CT scan after EVT. The presence of hemorrhagic transformation was defined by dividing into 4 groups: Type 1 hemorrhagic transformation is the formation of petechiae at the edges of the infarct. Scattered petechiae within the infarct area without any mass effect is defined as type 2. Parenchymal hemorrhage resulting in a mass effect with focal blood collection covering < 30% of the infarcted area is defined as type 3. Type 4 corresponds to hemorrhages that cover more than 30% of the infarcted area and/or create a significant space-occupying effect.

### 2.2. Laboratory analysis and inflammatory score calculation

The outcomes of EVT were evaluated according to the thrombolysis grading scale in cerebral infarction.^[12]^ Functional outcomes were assessed using the modified Rankin Scale score at discharge from the hospital. A poor clinical outcome was defined as an mRS of 3 or more.

Inflammatory indices were calculated using venous samples taken upon patient admission. Before the intervention, blood was drawn from the antecubital vein and collected in the appropriate tubes. Serum levels of neutrophils, lymphocytes, platelets, hemoglobin, C reactive protein (CRP), and albumin were measured along with lipid profiles as well as liver and kidney function parameters. These assessments were conducted using the Beckman UniCel DxC 800 Synchron Autoanalyzer (Beckman Coulter Inc., Brea) with the appropriate collection tubes.

Serum creatinine measurements were performed at baseline (before thrombectomy) and 48 to 72 hours after contrast medium use. CI-AKI was defined as an increase in serum creatinine levels more than 0.5 mg/dL or 25%.^[13]^

Demographic characteristics as well as laboratory and clinical data were compared between the groups with and without CI-AKI.

SII was calculated with the formula of: peripheral (platelet count × neutrophil count)/ lymphocyte count; SIRI was calculated with the formula of: (neutrophil count × monocyte count)/ lymphocyte count.^[14]^ Their predictive value for CI-AKI occurrence was compared.

### 2.3. Statistical analysis

Statistical analyses were conducted using SPSS, version 29.0, (IBM, SPSS Inc. Chicago). Data are expressed as mean ± SD for continuous variables and percentage for categorical variables. The Shapiro–Wilk test was used to test normality, and a *P*-value > .05 was defined as normally distributed data. Continuous variables that showed normal distribution were compared using Student *t* test. Mann–Whitney -tests were used for non-normally distributed samples. Associations of the categorical variables between groups were tested using the chi-square or Fisher exact test. Covariates were selected based on standardized differences between groups and their expected clinical impact on the outcome. To identify the factors associated with CI-AKI, we performed binary logistic regression analysis after adjusting for age, diabetes mellitus (DM), baseline renal function, cerebral edema, baseline NIHSS, body mass index (BMI), timing, contrast volume, and significant factors covariate effect analysis. ROC curve analysis was used to evaluate the discriminative power of 4 inflammatory indices: Platelet-to-Lymphocyte Ratio (P/L), Systemic Inflammatory Response Index (SIRI), Systemic Immune-Inflammation Index (SII), and NLR. The area under the curve was calculated for each index to measure diagnostic accuracy along with 95% confidence interval (CI) for precision. ROC curves were plotted to illustrate the sensitivity and 1-specificity offering a clear comparison of their performance. The optimal cutoff value for SII was determined using the maximum Youden index. All statistical analyses used 2-sided tests with a significance level (alpha) of 0.05.

## 3. Results

Among the 281 patients included in this study, CI-AKI was detected in 54 patients (19%). Patients who developed CI-AKI were significantly older (71.0 ± 8.8 vs 64.4 ± 13.3, *P* = .001), had a higher BMI (27.8 ± 4.6 vs 26.5 ± 3.9, *P* = .036), and a higher prevalence of DM (64.8 vs 42.3%, *P* = .004) (Table 1).

**Table 1:** Demographic and baseline data.

	CIN (−) n* = 227*	CIN (+) n* = 54*	*P*-value*
Age, yr	64.4 ± 13.3	71.0 ± 8.8	**.001**
Gender, female, n (%)	96 (42.3)	28 (51.9)	.224
BMI, kg/m^2^	26.5 ± 3.9	27.8 ± 4.6	**.036**
Obesity, n (%)	51 (22.5)	15 (27.8)	.475
Coronary artery disease, n (%)	82 (36.1)	23 (42.6)	.434
Prosthetic heart valve, n (%)	18 (7.9)	1 (1.9)	.138
Prior atrial fibrillation, n (%)	72 (31.7)	15 (27.8)	.626
Diabetes mellitus, n (%)	96 (42.3)	35 (64.8)	**.004**
Hypertension, n (%)	132 (58.1)	30 (55.6)	.729
Previous stroke or TIA, n (%)	63 (27.8)	22 (40.7)	.071
Current smoker, n (%)	15 (6.6)	5 (9.3)	.555
Chronic kidney disease, n (%)	17 (7.5)	8 (14.8)	.109
Congestive heart disease, n (%)	58 (25.6)	18 (33.3)	.306
CTA (+)	28 (12.3)	4 (7.4)	.306
Laboratory parameters			
Glucose, mg/Dl	128 ± 46	162 ± 69	**<.001**
Urea, mg/Dl	39.9 ± 18.9	53.1 ± 32.3	**<.001**
Creatinine, mg/Dl	0.9 ± 0.6	1.0 ± 0.3	.247
GFR, ml/min/1.73 m^2^	86 ± 25	70 ± 23	**<.001**
CRP, mg/L	5.9 (2.5–15.6)	5.4 (3.3–13.2)	.820
LDL-C, mg/Dl	112.8 ± 31.9	117.3 ± 41.2	.381
HDL-C, mg/Dl	45.5 ± 12.6	43.9 ± 10.7	.385
Triglyceride, mg/Dl	100.2 ± 60.7	98.4 ± 49.3	.838
WBC, 10^3^/µL	9.13 ± 2.58	10.10 ± 2.97	**.017**
Lymphocyte,10^3^/µL	1.7 ± 0.9	1.3 ± 0.5	**.001**
Hemoglobin, g/Dl	12.72 (1.93)	12.45 (1.79)	.352
Monocyte,10^3^/µL	0.7 ± 0.4	0.7 ± 0.4	.597
Neutrophil,10^3^/µL	6.48 ± 2.37	7.79 ± 2.66	**<.001**
Platelet count, 10^3^/µL	227.67 (45.18)	243.81 (58.56)	**.027**
Platelet/ Lymphocyte ratio	162.38 (72.45)	222.38 (112.45)	**<.001**
SII	1012 (559–1490)	1525 (1058–2082)	**<.001**
SIRI	2.52 (1.47–4.20)	3.86 (2.40–5.19)	**<.001**
Neutrophil/lymphocyte ratio	4.64 ± 2.41	7.10 ± 3.77	**<.001**
Clinical parameters			
*Cerebral Edema*, n (%)	52 (22.9)	28 (51.9)	**<.001**
Baseline NIHSS	17.55 (3.88)	19.30 (2.80)	**.002**
*NIHSS, post-op*	10.4 ± 6.9	18.7 ± 6.1	**<.001**
ASPECT	9.1 ± 0.9	8.9 ± 0.9	.208
Time to recanalization, min	53.2 ± 17.0	61.9 ± 21.1	**.003**
Onset of symptom, min	126.9 ± 84.2	150.6 ± 73.0	.058
Intraarterial tPA, n (%)	20 (8.8)	4 (7.4)	.740
Intravenous tPA, n (%)	5 (2.2)	1 (1.9)	.873
mRS at discharge	2.8 ± 2.0	5.0 ± 1.7	**<.001**
Hemorrhagic transformation, n (%)			
Absent	168 (74.0)	22 (40.7)	**<.001**
Petechial hemorrhages type I	15 (6.6)	4 (7.4)	
Petechial hemorrhages type II	21 (9.3)	5 (9.3)	
Parenchymal hematoma type I	12 (5.3)	10 (18.5)	
Parenchymal hematoma type II	11 (4.8)	13 (24.1)	
Contrast volume, ml	147 ± 24	157 ± 22	**.008**

Data are shown in n (%), median (interquartile range; 25th–75th percentiles), and mean ± standard deviation.

ASPECT = Alberta stroke program early CT score, BMI = body mass index, CIN = contrast-induced nephropathy, CRP = C reactive protein, CTA = computerized tomography angiography, GFR = glomerular filtration rate, HDL-C = high-density lipoprotein cholesterol, LDL-C = low-density lipoprotein cholesterol, mRS = Modified Rankin Scale, NHSS = National Institutes of Health Stroke Scale, SII = Systemic immune-inflammation index, SIRI = Systemic Inflammation Response Index, TIA = transient ischemic attack, tPA = tissue plasminogen activator, WBC = white blood cell

* A *P*-value of <.05 was considered statistically significant.

**Figure 1.**
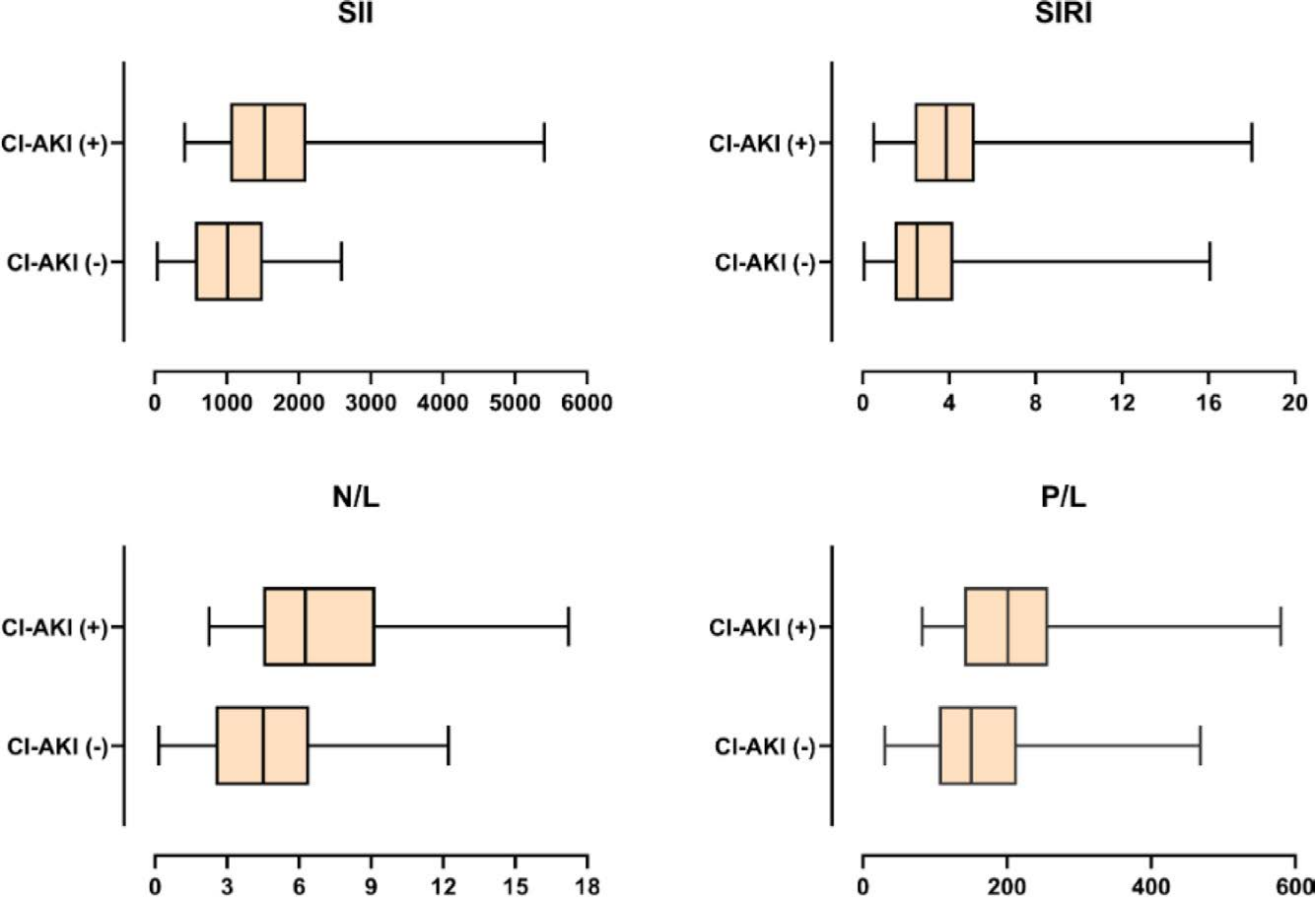
Box plots represent the distribution of systemic immune-inflammation index (SII), systemic inflammatory response index (SIRI), neutrophil-to-lymphocyte ratio (NLR), and platelet-to-lymphocyte ratio (P/L) in CI-AKI positive (CI-AKI+) and CI-AKI negative (CI-AKI−) patients.

Among laboratory parameters, glucose (162 ± 69 vs 128 ± 46 mg/dL), urea (53.1 ± 32.3 vs 39.9 ± 18.9 mg/dL), white blood cell count (10.10 ± 2.97 vs 9.13 ± 2.58 × 10³/µL), neutrophil count (7.79 ± 2.66 vs 6.48 ± 2.37 × 10³/µL), platelet count (243.81 ± 58.56 vs 227.67 ± 45.18 × 10³/µL), and inflammatory indices (SII: 1525 vs 1012, *P* < .001; SIRI: 3.86 vs 2.52, *P* < .001) were all significantly higher in patients with CI-AKI (Fig. 1). Similarly, the NLR (7.10 ± 3.77 vs 4.64 ± 2.41, *P* < .001) and platelet-to-lymphocyte ratio (222.38 ± 112.45 vs 162.38 ± 72.45, *P* < .001) were also elevated (Table 1).

Regarding clinical parameters, patients with CI-AKI had significantly higher baseline NIHSS (19.30 ± 2.80 vs 17.55 ± 3.88, *P* = .002), postthrombectomy NIHSS (18.7 ± 6.1 vs 10.4 ± 6.9, *P* < .001), cerebral edema (51.9% vs 22.9%, *P* < .001), recanalization time (61.9 ± 21.1 vs 53.2 ± 17.0 minutes, *P* = .003), and contrast volume (157 ± 22 vs 147 ± 24 mL, *P* = .008). Patients with CI-AKI also had worse functional outcomes at discharge (mRS: 5.0 ± 1.7 vs 2.8 ± 2.0, *P* < .001) and higher rates of hemorrhagic transformation (*P* < .001) (Table 1).

In multivariable logistic regression analysis, baseline glomerular filtration rate, baseline NIHSS, cerebral edema, recanalization time, SII and SIRI variables were found to be independent predictors of CI-AKI occurrence (Table 2).

**Table 2:** Univariable and multivariable logistic regression analysis for occurrence of CIN in acute stroke patients undergoing EVT.

Variables	Covariate effect	Group effect	OR (95% CI)†
	OR (95% CI)	OR (95% CI)*	
Age, per yr	1.05 (1.02–1.08)	1.03 (0.99–1.07)	1.02 (0.98–1.06)
Diabetes mellitus, yes	2.51 (1.36–4.66)	1.33 (0.61–2.90)	1.25 (0.55–2.84)
Baseline GFR, ml/min/1.73m^2^	0.97 (0.96–0.99)	0.98 (0.97–1.00)	0.98 (0.96–1.00)
Cerebral edema*, yes*	3.62 (1.96–6.71)	3.60 (1.66–7.81)	3.93 (1.73–8.96)
Baseline NIHSS	1.19 (1.06–1.33)	1.17 (1.02–1.34)	1.20 (1.03–1.38)
Time to recanalization, min	1.03 (1.01–1.04)	1.03 (1.00–1.07)	1.04 (1.01–1.07)
Contrast volume, ml	1.02 (1.01–1.03)	0.99 (0.97–1.02)	0.99 (0.96–1.01)
Body mass index, kg/m^2^	1.07 (1.00–1.15)	1.02 (0.94–1.12)	1.00 (0.92–1.09)
SII	1.00 (1.00–1.00)	–	1.00 (1.00–1.00)
SIRI	1.17 (1.06–1.30)	1.22 (1.06–1.39)	–

CIN = contrast-induced nephropathy, GFR = glomerular filtration rate, NIHSS = National Institutes of Health Stroke Scale, OR = Odds Ratio, SII = Systemic immune-inflammation index, SIRI = Systemic Inflammation Response Index.

* Statistical performance; Hosmer-Lemeshow test *P*-value=.832, Nagelkerke R square=0.325, −2 Log likelihood=185.9, Model Chi-square=56.5.

† Statistical performance; Hosmer-Lemeshow test *P*-value=.178, Nagelkerke R square=0.400, -2 Log likelihood=170.8, Model Chi-square=71.6.

**Figure 2.**
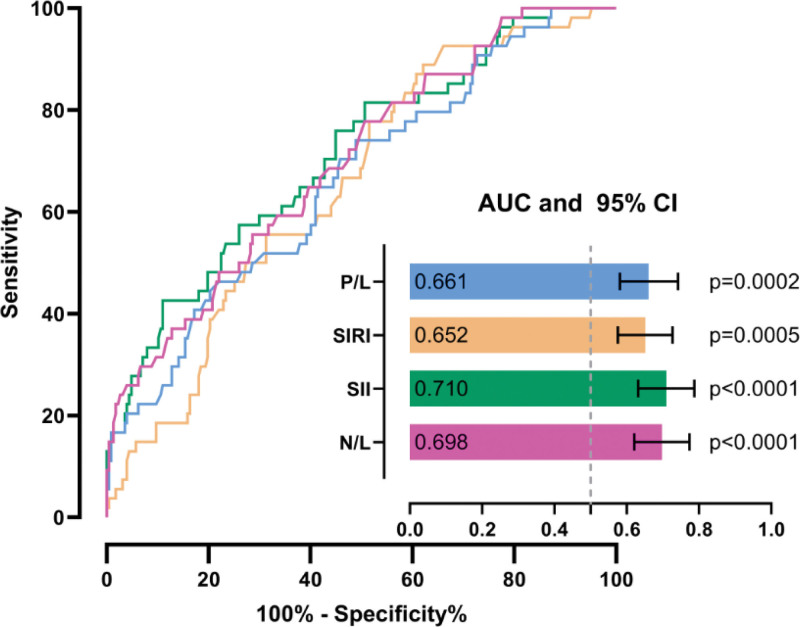
ROC curves show the diagnostic performance of 4 inflammatory indices – P/L, SIRI, SII, and N/L – for predicting contrast-induced acute kidney injury. area under the curve (bars) values and 95% CI (whiskers) are displayed for each index. NLR = neutrophil-to-lymphocyte ratio, PLR = platelet-to-lymphocyte ratio, SII = systemic immune inflammation index, SIRI = systemic inflammation response index.

Receiver operating characteristic curve analysis revealed that among inflammatory parameters, SII demonstrated the highest discriminative performance in predicting CI-AKI with a threshold of > 1784 (AUC = 0.710, 95% CI: 0.631–0.788, *P* < .001, sensitivity: 42.6%, specificity: 89.0%), followed by NLR (AUC = 0.698, 95% CI: 0.621–0.775, *P* < .001, sensitivity: 77.8%, specificity: 49.3%), PLR (AUC = 0.661, 95% CI: 0.580–0.742, *P* < .001, sensitivity: 74.1%, specificity: 51.1%), and SIRI (AUC = 0.652, 95% CI: 0.576–0.728, *P* < .001, sensitivity: 88.9%, specificity: 37.9%) (Fig. 2).

## 4. Discussion

### 4.1. The key findings of our study can be summarized as follows:

*(i*) Among clinical and procedural variables, baseline glomerular filtration rate, baseline NIHSS score, the presence of cerebral edema, and time to recanalization were independently associated with the development of CI-AKI. Additionally, both SII and SIRI were found to be significant predictors. *(ii*) Inflammatory markers, including PLR, NLR, SII, and SIRI, were significantly elevated in patients who developed CI-AKI compared to those who did not. Among these indices, SII demonstrated the highest discriminative ability for predicting CI-AKI, as shown by ROC curve analysis. Although age, DM, BMI, and contrast volume were higher in the CI-AKI group, multivariable logistic regression analysis showed that these variables were not independently associated with CI-AKI occurrence.

The occurrence of CI-AKI has been associated with long hospitalizations and increased mortality and morbidity.^[15]^ Although fewer nephrotoxic agents are currently used as contrast dyes, the risk of CI-AKI continues to be a significant problem. CI-AKI is also the third leading cause of acute renal failure in hospitalized patients.^[15]^ Considering the increased negative outcomes after CI-AKI, preventive measures are very important. Identifying patients at high risk for CI-AKI is perhaps the most important part of prevention. The insignificant amount of contrast medium administered may be related to the fact that patients generally received the standard minimum amount. Although the literature shows a strong correlation between the amount of contrast medium used and the development of CI-AKI, some studies have shown that this is not the only determinant.^[16,17]^ The patient’s general condition, renal function, and other underlying diseases should also be considered. In addition, previous renal dysfunction, DM, advanced age, decreased intravascular volume, female gender, history of previous vascular disease and nephrotoxic drug interactions have also been suggested to increase the risk of CI-AKI.^[18]^ Although DM is an important predisposing factor in most studies, there are also studies suggesting that CI-AKI is not triggered in the absence of diabetic nephropathy.^[19]^ DM was not found to be an independent risk factor in our study. The fact that advanced age and BMI were not found to be independent risk factors may suggest that our patient group should be evaluated together with the presence of other risk factors. The incidence of CI-AKI is highly influenced by type and volume of contrast agents, individual patient factors, or other determinants such as hemodynamic stability, systemic and renal perfusion dynamics, rather than the type of treatment procedure. However, the urgency of the treatment procedure is an important factor for CI-AKI development because preventive measures are the most effective element in minimizing CI-AKI.

Various pathophysiological mechanisms have been suggested for occurrence of CI-AKI. Contrast medium has a direct cytotoxic effect on renal tubular epithelial cells by increasing the formation of reactive oxygen species. The contrast medium can also induce vasoconstriction leading to hypoxia in the outer medulla thus further increasing the formation of reactive oxygen species via ischemia-reperfusion injury.^[20]^ Vasoconstriction induced with contrast agents also contributes to cell damage via hypoxia-associated necrosis. These possible mechanisms aggravate each other, exponentially increasing their damaging effects.

ROS also affect renal microcirculation by modulating vasoactive substances such as endothelin, adenosine, and nitric oxide.^[21]^ Therefore, a disruptive vicious cycle is created that leads to tubular cell death and associated inflammatory response.^[22]^ Inflammatory markers such as NLR, PLR, and CRP were also identified as predictors of CI-AKI occurrence after interventional procedures.^[6–8]^ SII and SIRI indexes reflect levels of inflammatory response. Recent studies have shown that high SII and SIRI scores can help predict outcomes in cardiovascular diseases.^[23,24]^ Huang et al^[25]^ found that increased SII scores were associated with poor short- and long-term clinical outcomes in patients with ST elevation acute coronary syndrome (STE-ACS) after primary percutaneous coronary intervention. Kelesoglu et al^[26]^ found SII to be an independent predictor of CI-AKI development in patients with non-ST ACS who underwent percutaneous coronary intervention. In a recent study, SII was found to be better predictor of CI-AKI than other inflammatory markers after carotid artery angiography.^[27]^ In this study, the cutoff value of SII to predict CI-AKI was 519.9 with a sensitivity of 80% and a specificity of 64%. In our study, when we used 1784 as the cutoff value of SII, it was found to predict CI-AKI with 42.6% sensitivity and 89.0% specificity.

Of course, MT is an urgent procedure, and there may be limited preventive options. Yamamoto et al^[28]^ found white blood cells count to be associated with a high risk of CI-AKI in patients who underwent EVT for AIS. SIRI is an inclusive indicator of chronic low-grade inflammation with measuring monocyte, neutrophil, and lymphocyte counts.^[10]^ Neutrophils are forerunner cells that reach the affected area after AIS. Although they have complex functions which are still not clearly defined, the neutrophil count in peripheral blood increases immediately after the onset of stroke. Higher neutrophil counts are associated with a larger infarct area and worse outcomes in several studies^[29,30]^ Monocytes reach the lesion after 24 hours. Monocytes are usually known to have a destructive role in the pathogenesis of acute stroke.^[31]^ The lymphocytes may play a contradictory role in acute stroke. While regulatory T cells are neuroprotective in early settings of ischemic stroke in mouse models, natural killer cells seem to be associated with late neuronal damage.^[32]^ In a recent study, SIRI was found to be an independent predictor of all-cause mortality in stroke patients.^[33]^ Although ROS-mediated tubular cell damage is the main pathophysiological mechanism in CI-AKI, the inflammatory response can also aggravate further cell damage. In addition to platelet/lymphocyte ratio, the neutrophil/lymphocyte ratio, and SII, our study also found that the SIRI is associated with CI-AKI. To the best of our knowledge, this study is the first to demonstrate the association of SIRI with CI-AKI.

### 4.2. Limitations

Our study has several limitations. As a single-center study, our patient population might be different from other centers. The size of our sample is relatively small, and our results need to be confirmed in future large multi-center prospective trials. Finally, we did not have the exact data regarding the amount of the contrast medium; nevertheless, we used an estimate while considering the number of angiography images.

## 5. Conclusion

The systemic immune inflammation index and systemic inflammation response indices were significantly higher in patients with CI-AKI. These scores may be new promising low-grade inflammatory indicators for predicting CI-AKI development.

## Author contributions

**Conceptualization:** Derya Ozdogru, Ilker Ozturk, Durmus Yildiray Sahin.

**Data curation:** Derya Ozdogru, Ilker Ozturk, Durmus Yildiray Sahin.

**Formal analysis:** Derya Ozdogru, Abdullah Yildirim, Ilker Ozturk, Durmus Yildiray Sahin, Zulfikar Arlier.

**Funding acquisition:** Derya Ozdogru, Abdullah Yildirim, Gulcin Yenice, Ilker Ozturk.

**Investigation:** Abdullah Yildirim, Gulcin Yenice, Ilker Ozturk, Zulfikar Arlier.

**Methodology:** Derya Ozdogru, Gulcin Yenice, Ilker Ozturk, Onur Kaypakli.

**Project administration:** Abdullah Yildirim, Gulcin Yenice, Onur Kaypakli.

**Resources:** Derya Ozdogru, Gulcin Yenice, Onur Kadir Uysal, Onur Kaypakli, Zulfikar Arlier.

**Software:** Abdullah Yildirim, Gulcin Yenice, Onur Kadir Uysal, Onur Kaypakli.

**Supervision:** Derya Ozdogru, Gulcin Yenice, Onur Kadir Uysal, Onur Kaypakli.

**Validation:** Abdullah Yildirim, Onur Kadir Uysal, Onur Kaypakli, Zulfikar Arlier.

**Visualization:** Derya Ozdogru, Onur Kadir Uysal, Onur Kaypakli, Durmus Yildiray Sahin.

**Writing – original draft:** Derya Ozdogru, Onur Kadir Uysal, Durmus Yildiray Sahin.

**Writing – review & editing:** Derya Ozdogru, Abdullah Yildirim, Onur Kadir Uysal, Durmus Yildiray Sahin, Zulfikar Arlier.
